# Embedding Scientific Explanations Into Storybooks Impacts Children’s Scientific Discourse and Learning

**DOI:** 10.3389/fpsyg.2020.01016

**Published:** 2020-06-10

**Authors:** Kathryn A. Leech, Amanda S. Haber, Youmna Jalkh, Kathleen H. Corriveau

**Affiliations:** ^1^School of Education, The University of North Carolina at Chapel Hill, Chapel Hill, NC, United States; ^2^Wheelock College of Education and Applied Human Development, Boston University, Boston, MA, United States

**Keywords:** book reading, explanations, parent–child interaction, scientific discourse, social interaction

## Abstract

Children’s understanding of unobservable scientific entities largely depends on testimony from others, especially through parental explanations that highlight the mechanism underlying a scientific entity. Mechanistic explanations are particularly helpful in promoting children’s conceptual understanding, yet they are relatively rare in parent–child conversations. The current study aimed to increase parent–child use of mechanistic conversation by modeling this language in a storybook about the mechanism of electrical circuits. We also examined whether an increase in mechanistic conversation was associated with science learning outcomes, measured at both the dyadic- and child-level. In the current study, parents and their 4- to 5-year-old children (*N* = 60) were randomly assigned to read a book containing mechanistic explanations (*n* = 32) or one containing non-mechanistic explanations (*n* = 28). After reading the book together, parent–child joint understanding of electricity’s mechanism was tested by asking the dyad to assemble electrical components of a circuit toy so that a light would turn on. Finally, child science learning outcomes were examined by asking children to assemble a novel circuit toy and answer comprehension questions to gauge their understanding of electricity’s mechanism. Results indicate that dyads who read storybooks containing mechanistic explanations were (1) more successful at completing the circuit (putting the pieces together to make the light turn on) and (2) used more mechanistic language than dyads assigned to the non-mechanistic condition. Children in the mechanistic condition also had better learning outcomes, but only if they engaged in more mechanistic discourse with their parent. We discuss these results using a social interactionist framework to highlight the role of input and interaction for learning. We also highlight how these results implicate everyday routines such as book reading in supporting children’s scientific discourse and understanding.

## Introduction

Although children rely on their own exploration and experimentation to learn about everyday scientific phenomena, this investigation alone is not always sufficient for children’s learning (Gelman et al., 2010; Harris and Corriveau, 2014; Legare, 2014). For example, when mechanisms that underlie a causal process are opaque or abstract, such as how electricity flows, children’s learning relies in part on information from others (Jipson et al., 2016; Harris et al., 2018). One type of information known to facilitate learning is adult explanations (e.g., Callanan and Oakes, 1992; Legare and Lombrozo, 2014; Lombrozo et al., 2018; Willard et al., 2019). However, as we describe in the text that follows, adults’ explanations to preschool-aged children vary in their frequency as well as in features that impact children’s learning (e.g., argument circularity, syntactic complexity, presence of causal mechanisms). Therefore, the primary aim of this study was to support adults in providing explanations that contain features that help children learn about abstract, unobservable scientific concepts. To do this, we designed a brief intervention in which features of scientific explanations were manipulated within a book-reading interaction between parents and their 4- to 5-year-old children. We investigated how such a manipulation impacted subsequent parent–child science interactions and children’s independent scientific thinking.

### Social Interactionist Theories of Learning

This study is motivated by social interactionist theories of development that state that children learn via input from and interaction with more knowledgeable others (Vygotsky, 1978; Bruner, 1983). Under this framework, interactions such as conversation between an adult and child support learning by helping the child organize knowledge and transfer such knowledge to novel situations. For example, talk about numbers relates to children’s early mathematical understanding (e.g., Ramani et al., 2015), discussions that contain spatial language relate to children’s spatial knowledge (e.g., Pruden et al., 2011), and references to emotions or mental states relate to children’s socio-emotional development (e.g., Lagattuta and Wellman, 2002; Ziv et al., 2013). Here, we explore how parent–child conversations about science support children’s scientific thinking and understanding. There are many features of science conversations that help children learn about scientific information, for instance, adults’ questions that highlight important information, connections to previous experiences, and explanations that elucidate unobservable scientific mechanisms (Beals and Snow, 1994; Snow and Kurland, 1996; Crowley and Siegler, 1999; Beals, 2001; Callanan and Jipson, 2001; Crowley et al., 2001a, b; Tenenbaum et al., 2005; Haden, 2010). The current article focuses specifically on the relation between explanations in parent–child conversation and children’s subsequent science learning.

### Parental Explanations Support Children’s Scientific Understanding

An explanation can be defined as talk that requests or makes a logical connection between objects, events, concepts, or conclusions (Beals, 1993). Explanations that meet this definition are relatively rare in everyday adult–child conversation (Rowe, 2012). Even when children ask questions that reference causal phenomena, parents respond with causal explanations only 50% of the time (Callanan and Oakes, 1992). In informal science settings, Kurkul et al. (unpublished) found that parents rarely produced explanations about electrical circuitry to their 4-year-old children unless prompted by a researcher. Additionally, Tabors et al. (2001) found that while interacting with their 5-year-old children around a magnet task, parents’ *science process talk*, a broad category that contained explanations, comprised only 14% of their utterances compared to 55% of utterances that referred to superficial qualities of the magnets.

Furthermore, when parents do provide explanations, there is variation in the extent to which the explanation contains *features* that support children’s learning (Baum et al., 2008; Russ et al., 2008; Corriveau and Kurkul, 2014; Mercier et al., 2014; Kurkul and Corriveau, 2018). The present study focuses on enhancing one feature of explanations: the presence of *mechanistic reasoning.* Mechanistic explanations, a form of causal explanations, provide information about the process through which a cause brings about an effect (Callanan and Oakes, 1992; Russ et al., 2008). Using a diary methodology, Callanan and Oakes (1992) found that parents most often used this type of explanation in response to 3- to 5-year-old children’s inquiries about causal events. Mechanistic explanations are argued to be central to children’s scientific theory-building because they highlight information that is not directly observable (Russ et al., 2008). Take, for instance, an explanation about how a light turns on. Although children may be able to observe a light turn on after a switch is flipped, they are not able to observe the process through which this occurs (i.e., electricity flows through the circuit to turn the light on). A more complete understanding can take place when an adult provides an explanation that elucidates the circuit mechanism.

Evidence that mechanistic explanations support children’s science learning comes from several previous studies. For example, Nolan-Reyes et al. (2016) found that explanations that contained mechanistic reasoning about impossible and improbable events predicted 4- and 6-year-old children’s possibility judgments and causal justifications for those judgments. Frazier et al. (2009) found that when experimenters provided 3- to 5-year-old children with mechanistic explanations, they were more likely to engage in subsequent information-seeking behaviors (i.e., asking follow-up questions) than when the experimenter provided a non-explanation. Studies with parents as the interlocutors also support the importance of mechanistic reasoning. For example, Willard et al. (2019) conducted a museum-based intervention study that encouraged parents to ask questions about gears in order to prompt mechanistic language (e.g., torque, motion) from their 4- to 6-year-old children (e.g., *How do the gears work?; What will happen when the gear moves?*). Parents who were assigned to the explanation condition had children who engaged in more scientific discourse and more often tested how the gears worked compared to a condition in which parents only encouraged children’s exploration. Kurkul et al. (unpublished) extended Willard et al. (2019) findings by showing that mechanistic conversations about circuitry between parents and 4-year-old children predicted children’s subsequent recall of the mechanistic explanation and their ability to transfer their knowledge to a novel scientific task.

Nevertheless, although children learn a great deal from explanations containing mechanistic reasoning, adults often struggle to adjust their language to provide accurate, yet developmentally appropriate mechanistic explanations, even if they understand the scientific mechanism under discussion (Gleason and Schauble, 1999; Schauble et al., 2002; Shtulman and Checa, 2012; Vlach and Noll, 2016). In the current study, we explored whether modeling mechanistic explanations using storybooks might increase parental use of these explanations to children during a subsequent informal science interaction.

To our knowledge, no study has examined whether parental explanations can be impacted via storybooks. However, other interventions without storybooks have proven effective and provide rationale for the current method. One strategy has been to provide parents with written instructions—termed “conversation cards”—for how to interact with their children (Fender and Crowley, 2007; Benjamin et al., 2010; Gutwill and Allen, 2010; Jant et al., 2014; Willard et al., 2019). In these studies, adult-child dyads or small groups of adults and children—mostly preschool and early elementary aged—receive different instructions for how to interact around a scientific exhibit. For example, Gutwill and Allen (2010) examined families’ interactions in a museum exhibit by comparing control families (who received no intervention) with families who received a scientific intervention known as “Juicy Questions,” which was aimed at increasing families’ use of questions, investigations, and discussions. Families who received the Juicy Questions intervention asked more questions and engaged in more inquiry-based exploration than families in the control condition (Gutwill and Allen, 2010). Additionally, Jant et al. (2014) presented dyads comprising parents and their 4-year-old children with conversational cards prior to visiting an exhibit, finding that dyads who received cards with elaborative questions (i.e., those that encouraged a multiword response from children) engaged in conversation containing more elaborative talk as compared with those dyads who did not receive conversational cards. Finally, Willard et al. (2019) found that parent-child dyads assigned to an explanation condition in which parents were encouraged to ask their child questions about how a set of gears works, engaged in more mechanistic conversation about gear functions. Moreover, such talk predicted children’s scientific exploration, operationalized as the testing of gears. Taken together, this research indicates it is possible to explicitly instruct adults to interact with children in certain ways (e.g., prompting parents to ask questions versus provide direct instruction or encouragement) in order to impact the scientific content of parent-child conversation and children’s independent learning.

A second, similar method conducted primarily in museum settings is to invite experts (e.g., experimenters, teachers, or museum educators) to model explanations for parents. For example, in Marcus et al. (2017), researchers demonstrated how to build skyscrapers to parents and 5- to 6-year-old children and then provided explanations about building engineering (e.g., what supports help make buildings strong). Dyads who received engineering explanations were able to transfer knowledge to a subsequent building activity compared to dyads who only received a building demonstration. Such scaffolded interventions work well in certain settings—such as on the museum floor or in the classroom where experts can join into dyadic interactions to scaffold learning. However, a constraint of this method is that parent-child interactions are dependent on initial modeling by an experimenter or teacher who is present. Instead, the present study sought to determine whether storybooks could replace experts in modeling the use of mechanistic explanations for parent-child interactions.

### Science Learning From Shared Book-Reading Interactions

Although typically examined in relation to early literacy outcomes (Fletcher and Reese, 2005) such as vocabulary (Wasik et al., 2016; Flack et al., 2018) and narrative ability (Zevenbergen and Whitehurst, 2003), book-reading interactions have also been used to examine the transmission of conceptual and scientific information between parents and preschool-aged children (Ganea et al., 2011), although note that this prior research did not examine transmission of mechanistic explanations. For example, manipulating book text to describe social categories using generic or non-generic language is associated with changes in parents’ subsequent essentialist language (Gelman et al., 2004; Rhodes et al., 2012; Chalik and Rhodes, 2015). Further, varying the linguistic structures of the story text (e.g., syntactically complex phrases, future tense) impacts 4-year-old children’s subsequent discourse and thinking (Vasilyeva et al., 2006; Leech et al., 2019a, b). Picture books hold a number of benefits for delivering scientific information to parent-child dyads. For example, the text is standardized to ensure accuracy of the scientific information, the content often contains information the dyad may not encounter frequently in the real world, and the setting provides an enjoyable situation where the parent and child share attention around an object (Ninio and Bruner, 1978; Kelemen et al., 2014). Previous work has described these scientific picture books as belonging to the *informational* or *expository* genre, which elicit more parent-child discourse, cognitively challenging questions, and opportunities to engage in reasoning than other genres (Duke, 2000; Anderson et al., 2004).

### Current Study

Exposing children to mechanistic explanations through book reading may be an ideal context for promoting scientific discourse because of the opportunity to standardize aspects of the text and as a result, transmit accurate scientific information to the dyad, just as experts do on the museum floor or in the classroom. This method may be especially useful for transmitting scientific information to parents who may be unsure of how to adapt this information for their preschool-aged children.

For this study, we focused on explanations about electricity and the mechanism that makes electricity work. We invited parent-child dyads to read one of two storybooks about electricity, one that contained mechanistic explanations (e.g., “*It is a kind of energy that makes things move, light up or get hot*”) or another that contained explanations that did not include mechanistic information (e.g., “*It’s a kind of energy that we can’t always see but it is very powerful*”). These non-mechanistic explanations were primarily analogical or those that connected the concept of electricity to prior knowledge, which have also been found to facilitate preschool children’s scientific understanding (Crowley and Jacobs, 2002; Valle and Callanan, 2006). Thus, dyads in both conditions read books that contained explanations that previous research has found to facilitate learning. After reading the book together, parents and children worked together during the *dyadic phase* to assemble electrical components of a circuit toy so that a light would turn on. Finally, during the *test phase*, children first independently assembled a novel circuit, and second, answered comprehension questions to gauge their understanding of electricity’s mechanism.

Our first research question asked whether reading storybooks containing mechanistic language leads to an increase in (a) parent-child use of mechanistic language and (b) children’s understanding of electricity’s mechanism measured during the *test phase*. We predicted that dyads in the mechanistic condition would use more mechanistic language than those in the non-mechanistic condition. We also predicted that children in the mechanistic would outperform children in the non-mechanistic condition on outcome measures in the *test phase*. Our second research question examined whether the frequency of mechanistic language produced by dyads would *strengthen* (i.e., statistically moderate) the effect of reading mechanistic storybooks on children’s scientific understanding. In line with social interactionist theories, conversations with more knowledgeable others help to organize children’s knowledge and allow the child to access it in subsequent situations (Vygotsky, 1978). On this theory, exposure to mechanistic explanations in storybooks alone may not be sufficient to teach children about the mechanisms underlying scientific concepts. Rather, we predicted that additional conversation between parents and children may help reinforce the mechanistic explanations modeled in the storybook, allowing the child to retrieve this knowledge during the *test phase*.

## Materials and Methods

### Participants

Seventy-six dyads were recruited to participate in the present study. Dyads were eligible for the study if they agreed to speak English during the study procedures and the child was between the ages of 4 and 6. Of the 76 dyads, we excluded *n* = 7 who were outside the age range and *n* = 8 who did not complete all procedures. This left a sample of 60 dyads for analysis. Note that *n* = 2 videos were not clear enough to transcribe and code conversation during the dyadic phase, leaving a sample of *n* = 58 for analyses that involve mechanistic conversation.

Our sample size was justified by an *a priori* power analysis using the G^∗^power program (Faul et al., 2009). We powered the study to answer our second research question (whether dyadic conversation moderates the effect of storybook condition on children’s scientific understanding) because the planned analyses required the most potential model parameters. The power analysis indicated that with 55 children, we have 0.80 power to detect a small effect size (*f*^2^ = 0.15) for the moderation effect using a multiple regression model.

Participating children (21 girls, 39 boys) were on average 60.3 months (*SD* = 5.99; range = 49.3–71.5 months). Three parents declined to provide a date of birth but identified the child as being within the age group required for the study. The majority of parents were highly educated, with 95% receiving at least a 4-year college degree (note, *n* = 6 parents declined to provide their educational background). Thirty percent of the parent sample (*n* = 17) self-identified as working in a STEM field, as measured by the question, “Do you consider your primary occupation to be in a STEM (science, technology, engineering, math) field? Yes or No.”

### Procedure

Dyads were recruited on the floor of a science museum, a laboratory, or preschools all located in a large northeastern city in the United States. All families were drawn from a similar population. Participant dyads were randomly assigned to one of two book-reading conditions: mechanistic (*n* = 32) or non-mechanistic (*n* = 28). Child age and sex were balanced across conditions. As compensation, participants at the museum were given a sticker and participants at schools and the laboratory were given a book. Data were collected between June 2018 and December 2018.

The study consisted of four phases in a fixed order: a *pretest* to gauge children’s preexisting understanding of electricity, a *book-reading interaction* during which dyads read their condition-specific storybook (mechanistic or non-mechanistic), a *dyadic phase* in which dyads completed a circuit task, and an individual child *test phase*. All phases were videotaped for later transcription, coding, and scoring. The entire procedure lasted approximately 15 min.

#### Pretest

Children were asked four questions about electricity in order to establish a measure of prior knowledge and to ensure the task was developmentally appropriate. First, children were asked: “*Have you ever heard of something called electricity?*” If the child answered yes, then the experimenter asked three follow-up questions: “*What do you think electricity is? Where does electricity come from? How does electricity travel?*” For the last two questions, the child was presented with a set of options, provided in pictorial form: “*Where does electricity come from?*” was asked with pictures of batteries, a faucet, and a planet (presented as outer space). “*How does electricity travel?*” was asked with pictures of wires, pipes, and a truck. The first two questions were asked in a fixed order; the last two questions were randomized.

#### Book-Reading Interaction

Parent-child dyads read a picture book containing explanations consistent with their condition. The experimenter provided the dyad with the condition-specific storybook and asked the parent to share the book with his or her child just like he or she would do at home. No time limit was given for completing the book and the average time spent on this phase was approximately 5 min.

A commercially available picture book, *Oscar and the Bird: A Book about Electricity* (Jeff Waring) was modified for the current study. Two versions of the text were prepared, which were matched on number of words, length of paragraphs, pictures, and linguistic complexity. Both books had 18 pages, 13 of which contained text and illustrations. The remaining five pages contained illustrations only. Books averaged 3.4 sentences per page. The critical difference between conditions was the content of the explanations. Each book contained eight explanations embedded into the text. For example, “*Electricity is a kind of energy that makes things move, light up, or get hot.*” The dyads in the non-mechanistic condition read a version in which the concepts were introduced in a procedural or analogical manner. For example, “*Electricity is a kind of energy that we can’t always see but is very powerful*.”

#### Dyadic Phase

**FIGURE 1:**
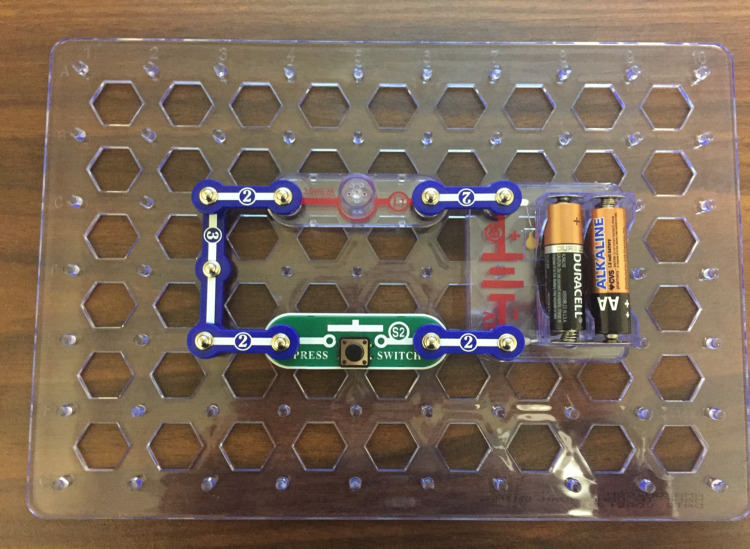
Example of completed circuit used in dyadic interaction.

Immediately following the book-reading interaction, the dyad was presented with a SnapCircuit© board and corresponding pieces (Figure 1). The procedure was identical across conditions. The child was first asked whether he or she had seen a toy like this before in order to take into account prior familiarity with the stimulus. Thirteen dyads (21.7% of sample) reported familiarity with the toy. The experimenter then said, “*You can put the pieces together and you can take them apart. Do you think you could the pieces together to make the light turn on?*” Dyads were given approximately 5 min to complete the task, after which the experimenter intervened and introduced the next phase. Stimuli consisted of seven components: blue snap wire pieces, a battery holder containing three 1.5 V AA batteries, a 2.5 V light, and a press switch.

#### Book Reading and Dyadic Phase Measures

Circuit completion was measured during the dyadic phase by whether the dyad successfully constructed the circuit to turn the light on. We also calculated the number of minutes the dyad spent assembling the circuit.

Second, we analyzed the conversations during the book reading and dyadic circuit phases to produce a measure of mechanistic discourse. First, all videos were transcribed at the level of the utterance by a team of research assistants according to the conventions of Child Language Data Exchange System (CHILDES; MacWhinney, 2000). Each transcript was then verified for accuracy by another trained research assistant. We then coded these transcripts for utterances that referenced mechanistic reasoning by adapting a coding scheme from Russ et al. (2008). Only extratextual speech (i.e., utterances that were not verbatim text) during the book-reading interaction was included in this measure. All mechanistic utterances during the dyadic phase were counted. Note that because mechanistic language was relatively rare, collapsing the two phases gave us more variation for the planned regression analyses. Reliability of two coders based on 15% of the transcripts resulted in 82% agreement (kappa = 0.80).

Our coding scheme captured different types of mechanistic language described by Russ et al. (2008): describing the target phenomenon (i.e., “*Make the light turn on*”); identifying entities by mentioning a circuit or connections (i.e., “*We have to connect the pieces in a circuit*”); identifying the activities and functions of the different entities (i.e., “*Electricity travels through the circuit to make the light turn on*”); and chaining backward (i.e., “*Why did the light turn on?*”) or forwards (i.e., “*If we took a piece off, would the light still turn on?*”) (Table 1). All utterances that fell into these categories were coded and summed to create a measure of mechanistic language. We chose to combine parental and child language together into one measure because we conceptualized the conversation as a co-construction of knowledge. For reference, Table 2 displays mechanistic talk for both speaking partners separately. This table shows that the proportion of mechanistic utterances is equivalent for parents and children, indicating that both speaking partners contributed to the conversation.

**TABLE 1:** Examples of mechanistic language during parent–child interaction.

**Category**	**Definition**	**Examples**
Describing target phenomenon	State phenomenon dyad is trying to produce	• We have to make the light turn on.
Identifying entities	Description of the enabling conditions that will make the mechanism run (i.e., light turn on)	• We have to connect the pieces in a circuit.
Identifying activities	The relevant activities that the entities engage in (functions of entities that cause changes in surrounding entities)	• Electricity travels through wires or the circuit. • Battery powers the circuit. • Switch controls the flow of electricity. • Wires connect the circuit.
Chaining backward/forward	A reasoning strategy that uses knowledge about the causal structure of the world to make claims about what must have happened previously (backward) or what will happen next (forward)	• Backwards: “Why did the light turn on?” • Forwards: “If we took a piece off, would the light still turn on?”

**TABLE 2:** Means, standard deviations, and ranges for mechanistic language variables separated by parent and child.

	**Parent**	**Child**
Mechanistic utterances	*M* = 9.90 (*SD* = 8.95; range = 0–35)	*M* = 1.26 (*SD* = 1.88; range = 0–9)
Total utterances	*M* = 160.31 (*SD* = 41.6; range = 83–258)	*M* = 25.69 (*SD* = 20.41; range = 1–117)
Proportion mechanistic utterances	*M* = 0.06 (*SD* = 0.05; range = 0–0.17)	*M* = 0.06 (*SD* = 0.10; range = 0–0.39)

Further, because time spent reading and interacting with the circuit varied across dyads, we created proportion measures by dividing dyads’ mechanistic utterances by the total number of utterances produced during the interaction.

#### Test Phase

This phase consisted of two tasks: an independent circuit task and comprehension questions. While the child completed this phase, the parent was given a short paper-and-pencil survey, which consisted of demographic items, reading habits at home, and feedback on the current study.

#### Test Phase Measures

To determine if the child could independently complete the circuit activity, the researcher presented the child with a novel circuit and seven circuit components: differently sized wire pieces, batteries, a sliding switch, and a fan. The child was given approximately 5 min to put the pieces together to make the fan turn on. If the child did not complete the task in 5 min, the researcher suggested moving on, and offered to help finish putting the circuit together at the end of the study. Children’s task completion was measured by their successful construction of a circuit that could turn the fan on within the time limit.

**FIGURE 2:**
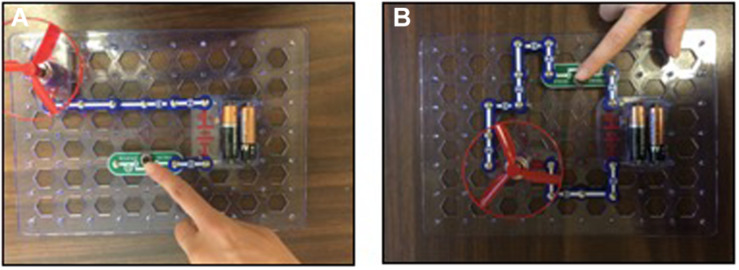
Children’s comprehension of electricity’s mechanism was probed by asking whether a disconnected circuit **(A)** and connected circuit **(B)** would make the fan go.

To measure children’s comprehension of electrical circuitry, the child was shown two pictures of a circuit similar to those used in the previous phases (Figures 2A,B). Figure 2A displays an unsuccessful mechanism (i.e., the circuit is disconnected) and Figure 2B displays a successful mechanism (i.e., a connected circuit). The child was asked: “*If I were to press the button in this circuit, do you think the fan would turn on? Yes or no? Why?*” Children were presented with one picture at a time and the order of presentation was randomized across participants. We coded the answers children provided to the posttest comprehension task by counting the number of correct answers (out of two) children earned (0 = incorrect; 1 = correct).

## Results

First, we ensured that there were no differences in demographic characteristics between the two conditions. Neither parent education (*p* = 0.67) nor STEM occupational status (*p* = 0.59) differed significantly between the mechanistic and non-mechanistic conditions. Similarly, family reading habits—the frequency with which parents reported reading informational books such as *Oscar and the Bird*—were similar across the two conditions, χ^2^ (*n* = 60, *df* = 4) = 7.11, *p* = 0.13. For reference, five parents reported reading informational books *hardly ever, n* = 9 reported *once or twice a month*, *n* = 37 reported *once or twice a week*, and *n* = 16 reported *almost daily.* We also examined children’s preexisting knowledge of electricity using pretest data, finding that 75% of children in the non-mechanistic and 84% of children in the mechanistic condition reported hearing about electricity, χ^2^ (*n* = 59, *df* = 1) = 0.72, *p* = 0.40. Finally, there was no significant condition difference in prior familiarity with the circuit stimuli, χ^2^ (*n* = 59, *df* = 1) = 0.54, *p* = 0.46.

### Do Mechanistic Storybooks Impact Dyadic Scientific Discourse?

Our first research question asked whether storybook condition led to differences in dyadic circuit assembly and mechanistic discourse. Looking first at the entire sample, *n* = 48 (80%) dyads successfully completed the joint circuit task. Importantly, condition differences emerged: nearly all dyads in the mechanistic condition (94%) constructed a circuit that turned on the light compared to 64% of dyads in the non-mechanistic condition, Fisher’s exact test (*n* = 60, *df* = 1), *p* = 0.008. Of those who finished the task, the average time to complete the circuit was 3 min, 40 s (*SD* = 1:40.56; range = 0:53–7:19). Mechanistic condition dyads (mean time = 3:18) were no faster at completing the task than non-mechanistic dyads (mean time = 3:59), *t*(44.18) = 1.51, *p* = 0.14 (corrected for unequal variance across groups).

We then explored condition differences in dyads’ use of mechanistic conversation. Collapsing both conditions (*n* = 58), dyads produced an average of 186.00 (*SD* = 52.40; range = 96–316) total utterances, of which 11.16 (*SD* = 9.65; range = 0–37) were coded as mechanistic. This corresponded to approximately 6% of dyads’ total talk. A significant difference in mechanistic language by condition emerged: the proportion of mechanistic utterances was significantly greater for dyads who read mechanistic storybooks (*M* = 0.07; *SD* = 0.05) than those who read non-mechanistic storybooks (*M* = 0.04; *SD* = 0.04), *t*(56) = 2.41, *p* = 0.01, Hedges’ *g* = 0.64. Note there was no significant difference in the *total* number of utterances produced within the mechanistic (*M* = 182.56; *SD* = 50.82) and non-mechanistic conditions (*M* = 190.23; SD = 54.99), *t*(56) = −0.55, *p* = 0.58, Hedges’ *g* = 0.15, suggesting that condition differences were isolated to mechanistic conversation only. Although dyads who completed the circuit (*M* = 0.06; *SD* = 0.05) used more mechanistic language than those who did not (*M* = 0.04; *SD* = 0.04), this difference was not statistically significant, *p* = *t*(56) = 1.41, *p* = 0.17. This lack of a statistical difference might be associated with the high percentage of dyads across both conditions (80%) who completed the joint circuit activity.

### Is There a Relation Between the Storybook Manipulation and Children’s Scientific Understanding?

We then examined the effects of condition on children’s scientific understanding during the *test phase*. When asked to independently construct a circuit, 55% of children (*n* = 33) succeeded in turning on the fan. We found that 69% of children in the mechanistic condition constructed a circuit that turned on the fan as compared to 39% of the children in the non-mechanistic condition, χ^2^ (*n* = 60, *df* = 1) = 5.24, *p* = 0.02. Children in the mechanistic condition (mean time = 3:20 min:s) were no faster at completing the circuit than children in the non-mechanistic condition (mean time = 3:33 min:s), *t*(55) = 0.49, *p* = 0.63. Further, children who succeeded at turning the fan on with their parents were more likely to have completed the dyadic circuit activity, χ^2^ (*n* = 60, *df* = 1) = 8.91, *p* = 0.003.

Next, we turned to children’s responses to the posttest comprehension items as a second index of their science understanding. Recall that comprehension was assessed by presenting children with pictures of a disconnected and connected circuit and asking them to reason about the outcome, that is, whether or not the fan would turn on. Forty-seven percent of children correctly answered that the disconnected circuit would not turn on, and 75% of children correctly answered that the connected circuit would turn on. An ordinal logistic regression model (Dependent Variable [DV] being 0 = neither picture correct, 1 = 1 picture correct, 2 = both pictures correct) indicated that children who read mechanistic storybooks were no more likely to correctly answer comprehension questions about the mechanism of electricity than children who read non-mechanistic storybooks, Estimate = 0.17 (*SE* = 0.49), Wald = 0.12, *p* = 0.73.

### Does Dyadic Mechanistic Conversation Strengthen the Relation Between Mechanistic Storybooks and Children’s Scientific Understanding?

Although there was no direct effect of Condition on children’s posttest comprehension, we hypothesized this effect may be statistically moderated by children’s social interactions with their parents. This hypothesis comes from our theory that language occurring during social interactions helps build children’s knowledge and transfer it to novel situations.

**FIGURE 3:**
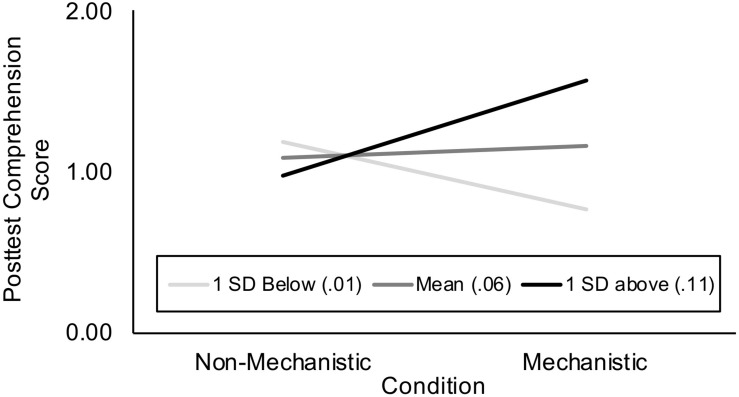
Mechanistic language moderates the effect of condition on children’s comprehension. Lines refer to the proportion of mechanistic utterances produced by dyads. Children in the mechanistic storybook condition were more likely to correctly answer the posttest comprehension questions only when they participated in higher levels of mechanistic conversation with their parents (black line).

We tested this hypothesis through a moderation analysis, regressing posttest comprehension scores on Condition, proportion of mechanistic utterances produced by the dyad (Mechanistic Conversation), and the interaction between the latter two terms (Table 3, model 1). We also controlled for whether the dyad successfully assembled the circuit (Dyadic Outcome) in order to isolate the effect of conversation. The overall model was significant, *R*^2^ = 0.19, *F*(4,52) = 3.07, *p* = 0.02. Our main interest was in the Condition × Mechanistic Conversation interaction, which was also significant, Δ*R*^2^ = 0.10, Δ*F*(1,52) = 6.13, *p* = 0.01, *b* = 10.85, *t*(52) = 2.48, *p* = 0.01. We probed the nature of the interaction by testing the conditional effect of Condition at three levels of mechanistic conversation: one standard deviation below the mean (proportion of utterances coded as mechanistic = 0.01), at the mean (0.06), and one standard deviation above the mean (0.10) (Figure 3). The Johnson–Neyman technique showed the relation between reading mechanistic storybooks and children’s success on the comprehension task was significant when dyads’ mechanistic talk comprised at least 11% of their total utterances during the circuit task. Thus, children in the mechanistic storybook condition were more likely to correctly answer the posttest comprehension questions when they participated in higher levels of mechanistic conversation with their parents. There was no such magnifying effect of mechanistic conversation for children who read storybooks with non-mechanistic explanations.

**TABLE 3:** Regression models showing moderated effects of condition on children’s science understanding.

**Variable**	**Posttest comprehension model 1**		**Independent circuit task model 2**	
		
	**Coefficient**	***t***	**Coefficient**	***z***
Intercept	1.35 [0.84, 1.87]	5.32***	−2.57 [−4.67, −0.47]	−2.40**
Dyadic outcome	−0.17 [−0.68, 0.34]	–0.67	2.03 [0.25, 3.80]	2.24*
Condition	−0.56 [−1.19, 0.07]	−1.79∼	1.60 [−0.38, 3.58]	–1.58
Mechanistic conversation	−2.26 [−9.37, 4.85]	–0.64	16.56 [−9.93, 43.05]	1.22
Condition × Mechanistic conversation	10.85 [2.06, 19.63]	2.48**	−18.34 [−49.52, 12.84]	–1.15
Model fit	*R*^2^ 19.1%		−2LL 66.80	

∼*p* < 0.10; * *p* < 0.05; ** *p* < 0.01; *** *p* < 0.001; dyadic outcome = whether dyad successfully completed the dyadic circuit task; brackets display 95% confidence intervals for model coefficients.

Our final analysis examined whether mechanistic conversation also moderated the condition effect on children’s independent circuit task performance. Table 3 (model 2) shows the results of a logistic regression predicting circuit success from Condition, Mechanistic Conversation, and the interaction between the latter two terms. This model fits better than a constant only model, χ^2^ (*df* = 4) = 12.98, *p* = 0.01. However, we did not find evidence that conversation served a moderating role, as indicated by a non-significant interaction effect, *b* = −18.34, *z* = 1.15, *p* = 0.25.

## Discussion

In this study, we examined how science storybooks may augment parent–child science conversations and children’s learning of a scientific mechanism. In particular, our first research question explored whether embedding explanations about causal mechanisms into a storybook about electricity could affect (a) parent–child talk about electrical mechanisms and (b) children’s individual understanding of such concepts. Drawing from social-interactionist theories, our second research question sought to determine whether mechanistic conversation produced spontaneously by parents and children magnified the effect of the storybook manipulation on children’s independent scientific understanding during the *test phase*.

### Does Embedding Explanations About Causal Mechanisms Into Storybooks Affect Parent–Child Science Understanding and Discourse?

Regarding (a) in the first research question, we found evidence that the book-reading manipulation was effective: dyads in the mechanistic condition were significantly more successful at completing a circuit task compared to dyads who read stories containing non-mechanistic explanations. Further, there was a significant difference in mechanistic language by condition, with dyads in the mechanistic condition using a greater proportion of mechanistic utterances compared to those in the non-mechanistic condition. Thus, it appears that providing verbal models of mechanistic reasoning via storybooks transfers to dyads’ joint understanding of scientific mechanisms as well as talk about such concepts.

To our knowledge, this is the first study to use storybooks to model mechanistic explanations about science concepts. We argue that embedding mechanistic language into the storybook text provided parents with a developmentally appropriate model to use with their children. As many parents may understand the concept of electrical circuitry but struggle to explain it to their preschool-aged children (Gleason and Schauble, 1999; Schauble et al., 2002; Shtulman and Checa, 2012; Vlach and Noll, 2016), storybooks may be an effective method for scaffolding parents’ explanations to children.

These results add to previous work showing how providing developmentally appropriate models of scientific explanations can help parents integrate this language into their conversations with children. For example, Jant et al. (2014) reported on a study where experimenters modeled explanations about building engineering, finding that modeling specific language for parents can modify the content of conversation and lead to deeper exploration and understanding of the topics at hand. Additionally, Benjamin et al. (2010) found that providing instructions prompting parents to ask more causal *wh-*questions during museum interactions was effective in boosting the use of such questions to children. The current study builds on this prior research by utilizing storybooks instead of museum educators or researchers as an interactive tool for prompting parental use of mechanistic language. Using a storybook method for modeling explanations may prove especially useful for scaling up parent–child interventions in the future, as a live experimenter or educator is not required to be present.

Moreover, most previous research on parent–child interventions around scientific discourse largely involves explicit instruction for parent–child interaction. For instance, previous studies have used conversational cards to explicitly instruct parents to ask more questions, be more elaborative, or encourage children’s exploration (Benjamin et al., 2010; Jant et al., 2014; and see Boland et al., 2003 for an example in a non-scientific setting). In the current study, we employed a less explicit method of storybooks to model a type of explanation known to facilitate learning. We argue that the storybook delivery method can be seen as a strengths-based approach to fostering scientific discourse. Storybooks containing embedded explanations provide parents with accurate scientific information while also allowing for considerable latitude in how the parent chooses to use the storybook text based on individual family dynamics or cultural values (e.g., Gutiérrez and Rogoff, 2003; Kline, 2015). For instance, a parent may choose to read the text verbatim, may augment the text with subsequent discussion, or employ a combination of these practices. The adoption of such a strengths-based approach holds important implications for adapting this study to other populations, as we discuss in more detail in the text that follows.

Interestingly, when examining the amount of mechanistic language produced in dyadic conversations, we found that it was quite rare, comprising approximately 6% of dyads’ total utterances. Though this proportion may appear small, it is consistent with prior research. For instance, Kurkul et al. (unpublished) found that parents do not often produce spontaneous mechanistic explanations to their children, and observational studies that have measured the frequency of child-directed explanations report similar proportions (Beals, 2001; Rowe, 2012). Though explanatory language is relatively rare in everyday conversation (Rowe, 2012), research has shown that even infrequent participation in such conversations is positively related to children’s scientific understanding (Tabors et al., 2001).

### Does Embedding Explanations About Causal Mechanisms Into Storybooks Affect Children’s Science Learning?

Regarding (b) in the first research question, we observed that storybook condition affected children’s learning during the independent circuit task. Here, children in the mechanistic condition were more successful at completing the circuit task than children in the non-mechanistic condition. These results show that not only does a book-reading manipulation affect parent–child scientific discourse, it also impacts children’s scientific behaviors on a different, but related task. This finding provides further evidence and extends Kurkul et al. (unpublished), where children’s performance on a similar circuit task was enhanced when they were systematically exposed to mechanistic explanations from experimenters. Here we show that using storybook interactions with parents is similarly effective. Because prior research has often looked solely at storybooks without a hands-on application task or solely at targeted games such as gears or circuits (Legare and Lombrozo, 2014; Kurkul et al., unpublished), our study extends this research by targeting both of these elements together.

Although we found that storybook condition influenced children’s performance on the independent circuit task, our results indicated that there was no main effect of condition on children’s responses to a series of comprehension questions gauging their mechanistic understanding. One explanation for this null result is that the comprehension questions relied on a deeper understanding of electricity’s mechanism than the independent circuit task. It is possible that in order to complete the independent circuit, children may have simply relied on their procedural memories, replicating what they did with their parent in the dyadic circuit task. However, in order for children to correctly answer the comprehension questions, they likely needed to possess an accurate understanding of the underlying mechanistic concept. On this hypothesis, children may have needed more scaffolding—such as participation in mechanistic conversation with their parents—to fully acquire an understanding of electricity’s mechanism.

Indeed, in our second research question, we found support for this hypothesis by showing that the effect of storybook condition on children’s comprehension was statistically moderated by children’s social interactions with their parents. The results indicate that mechanistic conversation amplified the effect of mechanistic storybooks on children’s science learning. We interpret these results using a social-interactionist framework: reading stories that contain mechanistic language may not be sufficient to teach children about the mechanisms underlying scientific concepts. Instead, we argue that social interaction serves as the process for learning and therefore, helped to strengthen the mechanistic concepts conveyed in the storybook. In other words, the more the dyad used mechanistic language, the better the child performed on the comprehension assessment.

This finding extends prior research and provides further evidence that social interaction is an important process by which adults influence children’s learning (Gunderson and Levine, 2011; Pruden et al., 2011; Marcus et al., 2017). We reached a similar conclusion to Jant et al. (2014) in that children learn about scientific mechanisms through a combination of “doing and talking.” Indeed, children can learn some of the information regarding electrical circuitry through hands-on contact with the circuit game. However, a more complete understanding of the mechanism underlying the circuit seems to require conversation and interaction with more knowledgeable others. This conclusion draws upon and unites two relatively separate literatures on how children learn, one of which focuses on children’s hands-on exploration (e.g., Piaget, 1964) and another that emphasizes the role of conversation and interaction (e.g., Vygotsky, 1978). Taken together, these findings indicate that systematic exposure to mechanistic explanations via storybooks coupled with opportunities to discuss and explore the mechanisms under question can be an effective way to improve children’s understanding of scientific concepts.

### Limitations

It is important to note that because data were collected primarily in a museum and laboratory context, the sample was drawn from a relatively educated, higher socioeconomic population of parents. One important direction for future research is to determine whether this manipulation would be successful with other populations of parent–child dyads, such as families from lower socioeconomic backgrounds. We argue that our storybook delivery method—as opposed to an explicit intervention with an experimenter—may potentially increase parents’ willingness to adopt the explanatory style modeled in the storybook. However, it is important to note that previous studies have found that higher socioeconomic status caregivers, particularly those with more education, report reading more frequently (Bus et al., 1995) and using more explanations with preschool-aged children (Rowe, 2012). These socioeconomic status differences in baseline patterns of parent–child interaction may suggest a need to modify the current procedures, by for example, providing more scaffolding during the book-reading interaction. Nevertheless, the application of a scientific storybook intervention across populations is an important question for future research.

Second, we chose to use parents rather than experimenters as children’s conversational partner to create a more naturalistic interaction. This design choice may have resulted in additional unexplained variation in features of conversation than if we used an experimenter. For instance, parents’ use of other linguistic features such as questions or non-verbal scaffolds may have also contributed to children’s science learning. However, because dyads were randomly assigned to the conditions and mechanistic explanations were the only manipulation, other conversational features that contribute to children’s learning should be equally distributed across the two conditions. Though examining potential condition differences in these features was beyond the scope of the study, we found no difference in dyads’ overall talkativeness (i.e., number of total utterances) across conditions. This gives us some evidence that it was the increase in mechanistic conversation, not other features of conversation, that caused improvements to children’s individual and joint learning.

Further, because parents did not implement the intervention in a standardized way (as an experimenter would have), there was wide variation in the amount of mechanistic language children heard, even within the same condition. This variation is evidenced by large standard deviations in the mechanistic language variables. Variation could possibly reflect that the “uptake” of the intervention was more successful for some dyads more than others. Future research with a larger sample size could test this claim by examining who this intervention works best for and why.

### Implications and Future Directions

There are several future directions that stem from this work. First, we focused on the concept of electricity and the mechanism that makes electricity work because it is developmentally appropriate for preschool-aged children and could be discussed during this single timepoint study. However, future studies may consider embedding other STEM concepts into storybooks, particularly those that are more opaque, such as germs, or more complex, such as forces and gravity. Another future direction is to determine the longitudinal effects of scientific storybooks on discourse and evaluate the longevity of the knowledge the children acquired during the intervention. As parents were asked to read the storybook with children on a single occasion, a next step could be to provide families with a set of books to take home and examine whether longer-term exposure leads to larger gains in children’s scientific understanding. Indeed, we know that repeated reading of storybooks leads to more extratextual talk and engagement from the adult and child (Robbins and Ehri, 1994; Senechal et al., 1995; Leung, 2008).

Our study has the potential to inform research on parent–child interaction and science learning. First, the results from this study demonstrate that subtle differences in how a concept is presented, namely, the presence or absence of mechanistic reasoning, can influence children’s learning. Recall that the illustrations, accuracy of information, linguistic complexity of text, and the length of the storybook explanations were equivalent across the two conditions. Thus, we are able to isolate changes in discourse and learning to the mechanistic manipulation. This finding holds implications for educators, parents, and other caregivers regarding the importance of integrating mechanistic reasoning into informal and formal educational settings. Second, storybooks may provide a window into science content that parents are less comfortable talking about with their child. Presenting scientific information in a storybook containing both narrative and informational elements may make the information more accessible, interesting and appealing to both parents and children, and is a cost effective and fun way to join learning with leisure. In considering formal learning contexts, this study can inform educators about ways to enhance science instruction across the curriculum. Research indicates that only 3–11% of preschoolers’ time is spent on science activities in early childhood classrooms versus nearly double the amount spent on literacy activities (La Paro et al., 2009; Piasta et al., 2014). Our results illustrate that literacy and science can be integrated, where children learn science content knowledge by engaging shared book reading. Indeed, shared reading of informational books is a practice that many educators utilize frequently.

## Conclusion

In sum, embedding mechanistic explanations in storybooks can be an effective way to increase children’s science discourse and learning. We present a framework for enhancing parent–child interactions that can be implemented in informal and formal learning settings by a variety of caregivers. Our findings add to the existing evidence that conversation between an adult and child plays an essential role in the development of mechanistic reasoning and more generally their understanding of science concepts during the early childhood period.

## Data Availability Statement

The datasets generated for this study are available on request to the corresponding author.

## Ethics Statement

The studies involving human participants were reviewed and approved by Boston University IRB. Written informed consent to participate in this study was provided by the participants’ legal guardian/next of kin.

## Author Contributions

KL, YJ, and KC designed the study. KL, AH, and YJ collected and processed the data. KL and AH analyzed the data and co-wrote the manuscript. KC and YJ provided feedback on manuscript drafts. All authors approved the final manuscript draft.

## Conflict of Interest

The authors declare that the research was conducted in the absence of any commercial or financial relationships that could be construed as a potential conflict of interest.
